# Glances at the Hospitals

**Published:** 1903-10-17

**Authors:** 


### GLANCES AT THE HOSPITALS.

### ROYAL BOSCOMBE HOSPITAL.

The total ordinary income was £1,457, and the total ordinary expenditure was £1,549. The average cost per bed occupied was £77 9s., and the average stay of each patient in the hospital was 24 days. The Governors say that a new operating theatre is much needed; and this must be the case, as the isolation ward is now being used as a theatre. The total number of in-patients treated during the year was 312. The medical, surgical and operation tables are properly drawn out, giving all necessary information as regards numbers and results. There were 264 operations. Ether was given 138 times, gas 96, A.C.E. mixture 74, chloroform 46, chloroform and ether 21, gas and ether 10, and gas, chloroform and ether 9. There was no death from any of these.

### ROYAL HOSPITAL FOR DISEASES OF THE CHEST, CITY ROAD, E.C.

The total income for the past year amounted to £7,254, and the ordinary expenditure to £7,308. It is proposed to build a nurses' home and a sanatorium for the open-air treatment of consumption, and for these purposes £50,000 will be required. On January 1st the hospital contained 70 patients, and 709 were admitted during the year, making a total of 779 under treatment. Of these 634 were discharged either cured or relieved, 75 died, and 70 remained in the wards at the end of the year. Over 11,000 patients were attended to at the out-patients' department. The report contains no medical statistics, hence it has no special medical interest.

